# METTL3 knockdown promotes the osteogenic differentiation of hPDLSCs by regulating CARD11 levels

**DOI:** 10.1016/j.clinsp.2025.100787

**Published:** 2025-10-14

**Authors:** Bing Zhou, Cheng Wang

**Affiliations:** Department of Stomatology, the 900th Hospital of Joint Logistic Support Force, Fuzhou, Fujian, China

**Keywords:** Periodontitis, METTL3, CARD11, hPDLSCs

## Abstract

**Objective:**

Periodontitis is a multifactorial progressive disease predominantly related to bacterial infection. Human Periodontal Ligament Stem Cells (hPDLSCs) are important cell populations for repair and regeneration in periodontal tissue. Recently, Methyltransferase-Like-3 (METTL3) is demonstrated to be closely related to the osteogenic differentiation of stem cells by modulating m6A levels. Additionally, through the bioinformatic analysis, CARD11 was demonstrated to be closely related to periodontitis. Therefore, this study was performed to investigate the role of METTL3 in the osteogenic differentiation of hPDLSCs and the periodontitis rat model.

**Methods:**

The biomarkers of hPDLSCs were analyzed by flow cytometry. qRT‒PCR was performed to measure METTL3 and CARD11 levels. The protein levels of Runx2, Osterix, Osteocalcin, p-p65, and p-IκBα were detected by western blotting. Alizarin red and alkaline phosphatase staining were carried out. The relationship between METTL3 and CRAD11 was confirmed by RIP and dual luciferase report.

**Results:**

The results showed that METTL3 and CARD11 were dramatically upregulated in periodontitis patients. In addition, METTL3 silencing dramatically decreased the m6A and mRNA levels of CARD11 and increased the Runx2, Osterix, and Osteocalcin levels. Alizarin red and alkaline phosphatase staining showed that METTL3 silencing promoted the osteogenic differentiation of hPDLSCs. Additionally, overexpression of CARD11 neutralized the role of CARD11 in hPDLSCs and inactivated the NF-κB signaling pathway. Finally, METTL3 knockdown relieved the periodontitis progression in vivo*.*

**Conclusions:**

In conclusion, METTL3 participates in periodontitis progression by regulating CARD11 levels through the NF-κB signaling pathway.

## Highlights

• METTL3 was upregulated in periodontitis.
• METTL3 silencing promoted the osteogenic differentiation of hPDLSCs.
• METTL3 regulated the m6A levels of CARD11.
• CARD11 overexpression neutralized the role of si-METTL3 in hPDLSCs.

## Introduction

Periodontitis is a multifactorial progressive disease predominantly related to bacterial infection. The pathogenesis and developmental process of periodontitis are complex and closely related to bacteria, bacterial products, oral cells and cytokines.^1^^,^^2^ Among the clinical manifestations of periodontitis, alveolar bone loss is the most serious.^3^ In recent years, accumulating studies have shown that periodontitis is related to genetic modification.^4^^,^^5^ Human Periodontal Ligament Stem Cells (hPDLSCs) are important cell populations for repair and regeneration in periodontal tissue.^6^ hPDLSCs have immunomodulatory properties and can differentiate into different cell types, such as adipocytes and osteoblasts., etc., which are considered to be the seed cells with the greatest potential for periodontitis treatment.^7^^,^^8^

N6 Methyladenosine (m6A) is a dynamic methylation located at the N6 site of adenosine, which was first discovered in the 1970s and is the most common internal modification in eukaryotic Mrna.^9^^,^^10^ In the m6A methyltransferase complex, Methyltransferase-Like-3 (METTL3) and Methyltransferase-Like-14 (METTL14) form a heterodimer,^11^ in which METTL3 has catalytic activity and METTL14 plays a supporting role in the structure.^12^ Recently, studies have demonstrated that METTL3 is closely related to the osteogenic differentiation of bone mesenchymal stem cells by modulating m6A levels.^13^ Zhang et al.^14^ demonstrated that METTL3 silencing suppressed osteoblast differentiation by stabilizing Smad7 and Smurf1 mRNA transcripts in LPS-induced inflammation. However, the role of METTL3 in the osteogenic differentiation of hPDLSCs remains unclear.

Caspase Activation and Recruitment Domain 11 (CARD11) is a scaffold protein composed of 1154 amino acids that belongs to the Membrane-Associated Guanylate Kinase (MAGUK) superfamily and plays an important role in intracellular signal transduction mediated by T-cell receptors and B-cell receptors.^15^^,^^16^ CARD11 site mutation is associated with persistent activation of NF-κB, with self-activation after mutation.^17^ The NF-κB signaling pathway was demonstrated to be involved in the osteogenic differentiation of hPDLSCs.^18^

Therefore, this study was carried out to explore the specific effects of METTL3 on the osteogenic differentiation of hPDLSCs. The authors hypothesized that METTL3 might participate in periodontitis progression by regulating CARD11 levels.

## Material and methods

### Bioinformatic tools

The differentially expressed genes in periodontitis were acquired from the Gene Expression Omnibus database (GSE173078). The heatmap and volcano map of these differentially expressed genes in periodontitis were obtained by using the preprocess Core package in R software. Moreover, enrichment analysis of the Kyoto Encyclopedia of Genes and Genomes (KEGG) pathway for DEGs was performed using the clusterProfiler package (Version 2.4.3).

### Patients

Normal periodontal ligament tissues were collected from patients (*n* = 31) aged 35.1 ± 4.5 years-old who underwent removal of their premolars or third molars (due to orthodontic or ectopic eruption of wisdom teeth) in the 900th Hospital of Joint Logistic Support Force. The periodontal ligament tissues of periodontitis patients (*n* = 31) were collected from patients with periodontitis in the 900th Hospital of Joint Logistic Support Force; these patients were 37.6 ± 5.3 years old. The inclusion criteria were as follows: 1) The number of residual teeth in the mouth was not <15. The affected teeth that need to be removed due to severe periodontitis had no obvious caries, pulp and periapical diseases; 2) Subjects did not receive periodontal treatment and did not take antibiotics within 6-months before treatment; 3) Bone loss was clearly observed on the X-Ray film of parallel projection, and there were at least 5 natural teeth in each quadrant except the third molar and third degree loose teeth; 4) The subjects had good plaque control and no surgical treatment. The exclusion criteria were as follows: 1) Systemic diseases affecting periodontal health and healing; 2) Pregnancy or lactation; 3) Alcoholism, heavy smoking (> 20 cigarettes/day) and other bad habits; 4) Diabetes and other systemic diseases; and 5) Penicillin drug allergy history. The clinical study was conducted according to the ARRIVE guidelines.

### Cell culture

hPDLSCs were isolated and cultured by the enzymolysis tissue block method and subcultured when the cell confluence reached 70 %. Third-generation hPDLSCs were collected and washed twice with PBS containing 2 % fetal bovine serum, and the cell concentration of hPDLSCs was adjusted to 1 × 10^5^ pieces/mL. Next, PE-labeled CD11b and CD45 and FITC-labeled CD90, CD105, and D146 antibodies were added to the cells and incubated at 4 °C in the dark for 30 min. Then, the cells were incubated. The cells were washed twice with PBS containing 2 % fetal bovine serum and identified by flow cytometry.

### Cell transfection

The small interfering RNA METTL3 (si-METTL3 1# and si-METTL3 2#) and si-NC and the CARD11 overexpression vector and empty vector were provided by Shanghai GenePharma Co., Ltd. (Shanghai, China). Next, hPDLSCs were transfected with si-METTL3 of the CARD11 overexpression vector with Lipofectamine 2000 reagent (Invitrogen, CA, USA).

### RT-qPCR

Cellular RNA was isolated from cells with TRIzol (Beyotime, Nantong, China). In addition, the concentration and purity of the separated RNAs were determined with a UV spectrophotometer. The separated RNAs were converted to cDNA with a reverse transcription kit (Vazyme, Nanjing, China). Next, the SYBR® Premix Ex Taq™ quantitative kit (Vazyme) was selected to carry out the PCR. The reaction conditions were as follows: 95 °C, 30 s; 95 °C, 5 s; 60 °C, 30 s, 40 cycles. With GAPDH as the internal reference, the relative expression was calculated with the 2^−ΔΔCt^ method. The primer sequences (5′ ‒ > 3′) were listed as follows.

METTL3, Forward Primer TTGTCTCCAACCTTCCGTAGT, Reverse Primer CCAGATCAGAGAGGTGGTGTAG; Runx2, Forward Primer GACTGTGGTTACCGTCATGGC, Reverse Primer ACTTGGTTTTTCATAACAGCGGA; Osterix, Forward Primer GATGGCGTCCTCTCTGCTT, Reverse Primer TATGGCTTCTTTGTGCCTCC; Osteocalcin, Forward Primer CCTGGCAGGTGCAAAGCCCA, Reverse Primer GGGGGCTGGGGCTCCAAGT; CARD11, Forward Primer GCTCACAACCGCATCCCAA, Reverse primer CTCCTCATGACCGCCATGTT; GAPDH, Forward Primer GGCACAGTCAAGGCTGAGAATG, Reverse primer ATGGTGGTGAAGACGCCAGTA.

### Alizarin red staining

The hPDLSCs were cultured in 35-mm plates for 14 days. Then, 5 % ethanol was selected to fix the hPDLSCs, and 1 % Alizarin Red S (ARS) staining solution was used to stain the hPDLSCs for 15 min. Finally, ImageJ 1.48 software was used to perform densitometric analysis to evaluate the mineralization levels.

### Alkaline phosphatase staining and activity

First, 4 % paraformaldehyde was used to fix the hPDLSCs at room temperature for 10 min. After that, the hPDLSCs were stained with ALP for 20 min. A scanner was utilized to capture the images. Next, the ALP activity was measured with an ALP Assay Kit provided by Nanjing Jiangcheng Bioengineering Institute (Nanjing, China).

### Detection of m6A levels of CARD11

A magna methylated RNA immunoprecipitation m6A Kit (Merck Millipore, Germany) was used for detecting the m6A levels of CARD11. Total RNA was randomly fragmented. Magnetic beads A/G were incubated with anti-m6A for 30 min to prepare anti-m6A magnetic beads. The fragmented RNA, RNase inhibitor, and IP buffer were mixed and incubated with anti-m6A magnetic beads at 4 °C for 2 h. After elution, RNA was purified, and mRNA expression was determined by RT-qPCR.

### RIP assay

For RNA Immunoprecipitation (RIP), approximately 2 × 10^7^ cells were harvested and lysed in RIP lysis buffer (50 mM Tris–HCl, pH 7.4, 150 mM NaCl, 1 mM EDTA, 0.5 % NP-40, 0.5 % Triton X-100, 1× protease inhibitor cocktail, and 100 U/mL RNase inhibitor). Cell lysates were centrifuged at 16,000×g for 10 min at 4 °C, and the supernatant was collected. Protein A/G magnetic beads (BersinBio, Bes5101) were pre-equilibrated with RIP lysis buffer and incubated with 5 μg of anti-METTL3 antibody or anti-CARD11 antibody, or normal rabbit IgG (negative control) at 4 °C for 2 h. The antibody-bead complexes were then incubated with cell lysates overnight at 4 °C with gentle rotation. After washing with RIP wash buffer (50 mM Tris–HCl, pH 7.4, 300 mM NaCl, 1 mM EDTA, 0.1 % NP-40), the bound RNA was eluted using RNA extraction buffer (1 % SDS and 0.1 M NaHCO_3_) and purified using the RNeasy Mini Kit (QIAGEN). The enriched RNA was reverse-transcribed into cDNA, and the association of METTL3 and CARD11 with target RNAs was validated by qRT-PCR with gene-specific primers. The relative enrichment was normalized to input RNA (5 % of the original lysate) and expressed as 2^^-ΔΔCt^^ values.

### Luciferase reporter assay

Through the SRAMP database (http://www.cuilab.cn/m6asiteapp/old), the authors obtained the two m6A modification sites (site 1#, 690; site 2#, 1769) of CARD11. To assess the effect of CARD11 site 1# and site 2# mutations on luciferase activity in hPDLSCs following METTL3 knockdown, a dual-luciferase reporter assay was performed. hPDLSCs were transfected with plasmids harboring CARD11 site 1# or site 2# mutants cloned into the psiCHECK-2 vector downstream of the Renilla luciferase gene. Twenty-four hours post-transfection, cells were infected with lentiviral particles carrying si-METTL3 or non-targeting control siRNA. After an additional 48 h, cells were harvested, and luciferase activity was measured using the Dual-Luciferase Reporter Assay System according to the manufacturer's instructions. Firefly luciferase served as an internal control for normalization. Relative luciferase activity was calculated as the ratio of Renilla to Firefly luciferase activity.

### Western blot

First, the authors separated the proteins from the cells with RIPA lysis buffer (Beyotime). Next, the separated proteins were tested for concentration with a BCA kit. After that, the authors selected 40 μL of protein to perform SDS-PAGE, and then, the proteins were transferred onto PVDF membranes. Subsequently, the membranes were treated with 5 % bovine serum albumin for one hour and incubated with primary antibodies (anti-Runx2, 1:800; anti-osterix, 1:1000; anti-osteocalcin, 1:600; anti-p-p65, 1:1500; anti-p65, 1:1000; anti-p-IκBα, 1:1000; anti-IκBα, 1:1000; anti-GAPDH, 1:2000; Abcam, CA, USA) for 12 h. Next, the membranes were incubated with the secondary antibody for 2 h. Finally, the bands were analyzed with an ECL kit (Sbjbio) with GAPDH as the internal reference.

### Animal experiments

A periodontitis model was induced in 8-week-old male Sprague-Dawley rats by ligature placement around the bilateral maxillary second molars. Sterile 4‒0 silk sutures were placed subgingivally and left in situ for 14 days to induce inflammatory bone loss. To achieve METTL3 knockdown, rats were randomly divided into two groups: control (shNC) and METTL3 shRNA (shMETTL3). One week prior to ligation, rats in the shMETTL3 group received local injections of lentiviral particles encoding METTL3-targeting short hairpin RNA into the periodontal tissues, while control animals received scrambled shRNA. The injection sites were approximately 1 mm apical to the gingival margin at three points around each target tooth, with a total of 10 μL virus suspension per site. After ligation, the animals were maintained for an additional 14 days with weekly reinjection of the viral solution. Maxillae were then collected for micro-CT analysis to analyze the Bone Volume/Total Bone Volume (BV/TV) and Bone Mineral Density (BMD). The METTL3 and CARD11 expression levels in periodontal tissues were confirmed by Western blot. The levels of TNF-α, IL-1β and IL-6 in periodontal tissues were detected by ELISA kits (Nanjing Jiangcheng Bioengineering Institute, Nanjing, China). All experimental procedures were approved by the Institutional Animal Care and Use Committee and followed ARRIVE guidelines.

### Statistical analysis

All data were assessed with SPSS 22.0 to analyze the significance of differences. All experiments were conducted independently three times (*n* = 3). The results are presented as the mean ± SD. Student’s *t*-test and ANOVA followed by Tukey test were selected for the significance analysis. Pearson correlation analysis was performed to analyze the relationship between METTL3 and CARD11. A p-value smaller than 0.05 indicated statistical significance.

## Results

### Characterization of hPDLSCs

**Fig. 1:**
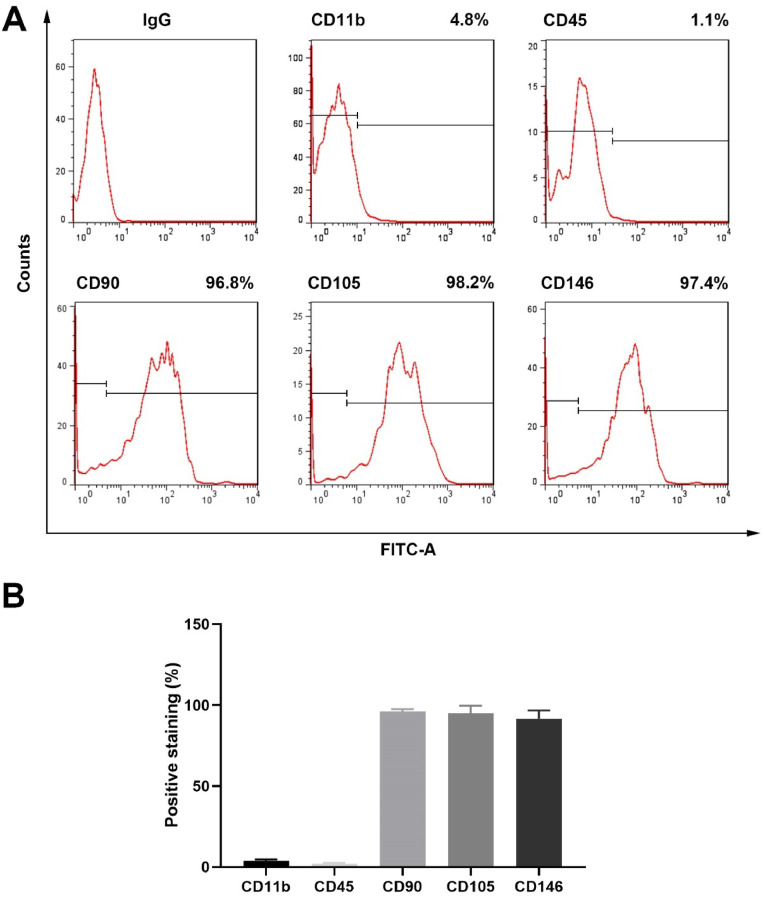
Characterization of hPDLSCs. (A) The positive markers CD90, CD105, and CD146, as well as the negative markers CD11b and CD45, of the hPDLSCs were measured by flow cytometry. (B) Quantification of flow cytometry results (n = 3).

To identify the hPDLSCs, the authors detected the expression of the positive markers CD90 (96.8 %), CD105 (98.2 %), and CD146 (97.4 %), as well as the negative markers CD11b (4.8 %) and CD45 (1.1 %) (Fig. 1A‒B).

### METTL3 was upregulated in periodontitis

**Fig. 2:**
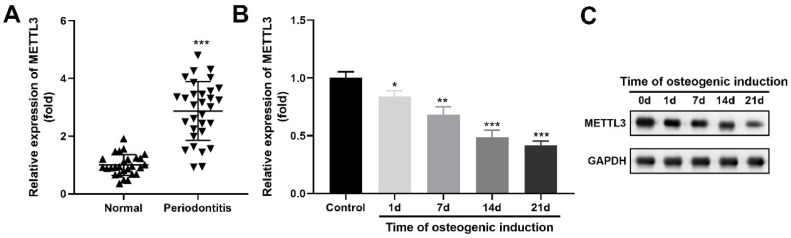
METTL3 was upregulated in periodontitis. METTL3 levels in periodontitis patients (n = 31, A) and hPDLSCs with osteogenic induction (n = 3, B) were determined by qRT-PCR. * p < 0.05, ** p < 0.01, *** p < 0.001.

METTL3 was dramatically upregulated in the periodontal ligament tissues of the periodontitis patients compared with those of the healthy controls (Fig. 2A). Besides, during the process of hPDLSCs osteogenic differentiation, METTL3 was downregulated in a time-dependent manner at both mRNA (Fig. 2B) and protein (Fig. 2C) levels.

### METTL3 silencing promoted the osteogenic differentiation of hPDLSCs

**Fig. 3:**
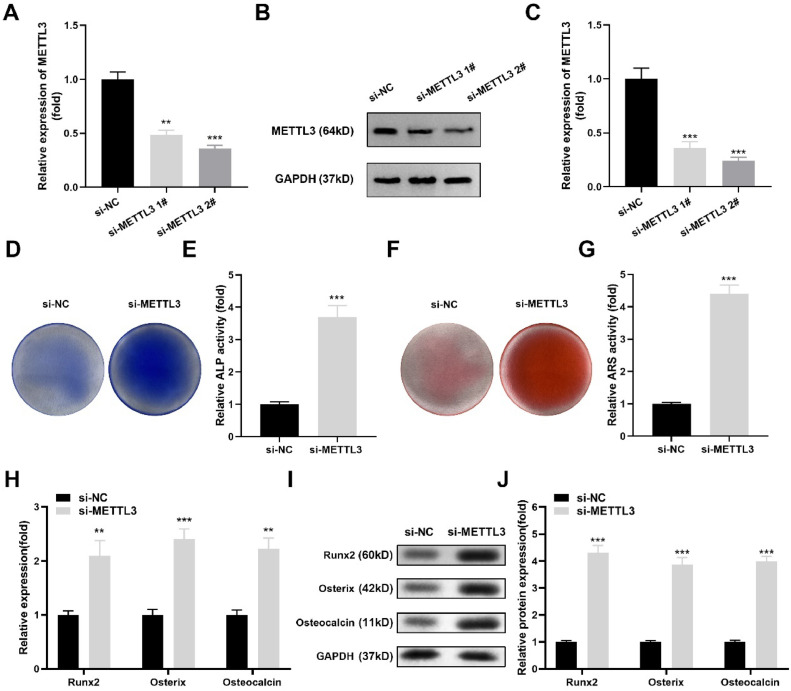
METTL3 silencing promoted the osteogenic differentiation of hPDLSCs. (A) RT‒qPCR and western blotting (B‒C) were performed to validate the transfection efficiency. (D‒E) ALP activity of hPDLSCs. (F‒G) ARS staining of hPDLSCs. (H) The mRNA levels of Runx2, Osterix and Osteocalcin were measured by qRT‒PCR. (I‒J) The protein levels of Runx2, Osterix and Osteocalcin were detected by western blotting (n = 3). ** p < 0.01, *** p < 0.001.

Next, the authors designed si-METTL3 to knock down METTL3 expression in hPDLSCs. After si-METTL3 transfection, the mRNA (Fig. 3A) and protein levels (Fig. 3B‒C) of METTL3 were significantly decreased. Among them, si-METTL3 #2 exhibited a better effect; therefore, the authors selected si-METTL3 #2 to perform the next experiments. After METTL3 silencing, ALP activity (Fig. 3D‒E) and red calcium nodules (Fig. 3F‒G) were prominently increased. Additionally, Runx2, Osterix and Osteocalcin were prominently upregulated at the mRNA (Fig. 3H) and protein (Fig. 3I‒J) levels after METTL3 silencing. To avoid off-target effects, the authors repeated these experiments using si-METTL3 #1 and obtained the same results (Supplementary Fig. 1).

### METTL3 affects the expression of CARD11 by regulating the level of m6A modification

**Fig. 4:**
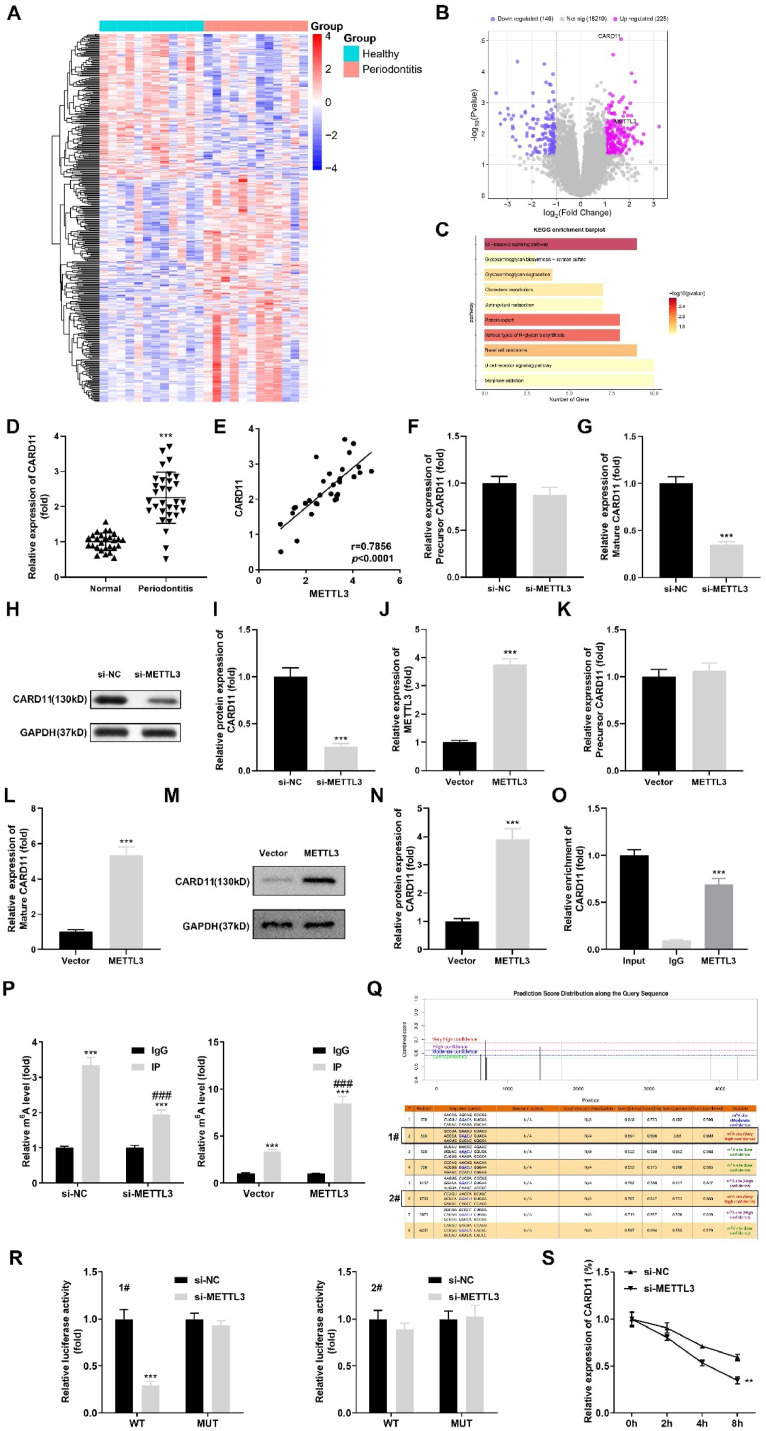
METTL3 affects the expression of CARD11 by regulating the level of m6A modification. The differentially expressed genes in periodontitis are expressed as a heatmap (A) and a volcano map (B). (C) KEGG pathway enrichment analysis of the differentially expressed genes in periodontitis. (D) The mRNA levels of CARD11 in periodontitis were tested by qRT-PCR. (E) Correlation analysis between CARD11 and METTL3 in periodontitis patients. The precursor (F) and mature (G) CARD11 levels were tested by qRT-PCR after METTL3 silencing. (H‒I) The protein expression of CARD11 was tested by western blotting after METTL3 silencing. (J) Validation of the transfection efficiency of the METTL3 overexpression vector. The precursor (K) and mature (L) CARD11 levels were tested by qRT-PCR after METTL3 overexpression. (M‒N) The protein expression of CARD11 was tested by western blotting after METTL3 overexpression. (O) RIP assay was performed to confirm the relationship between METTL3 and CARD11 (P). The m6A levels of CARD11 were detected by MeRIP assay after METTL3 silencing and overexpression. (Q) The M6A modification sites of CARD11 were predicted by SRAMP database, and the stability of CARD11. (R) Luciferase activity of CARD11 was detected after METTL3 silencing. (S) The mRNA stability of CARD11 was detected by RT-qPCR after METTL3 silencing (n = 3). ** p < 0.01, *** p < 0.001, ### p < 0.001.

Subsequently, according to the bioinformatic results, 225 upregulated genes and 146 downregulated genes in periodontitis were obtained and are shown in the heatmap (Fig. 4A) and volcano map (Fig. 4B). Additionally, through KEGG analysis, the authors found that the DEGs were enriched in many signaling pathways. Among them, compared to other pathways, the NF-kappa B signaling pathway was more closely related to periodontitis. In addition, the authors found CARD11 was one of the genes enriched in the NF-kB pathway, and its research in periodontitis is currently very limited. So CARD11 and NF-kappa B pathways were selected for further research (Fig. 4C). Then, the authors determined the CARD11 levels in periodontitis patients and found that they were prominently increased (Fig. 4D). The Pearson analysis indicated that CARD11 was positively related to METTL3 in periodontitis patients (Fig. 4E). After METTL3 silencing, mature CARD11 mRNA expression was prominently downregulated, while precursor CARD11 was not affected (Fig. 4F‒G). In addition, the protein levels of CARD11 were significantly decreased after METTL3 silencing (Fig. 4H‒I). Besides, METTL3 overexpression vector transfection increased the METTL3 mRNA expression levels of METTL3 (Fig. 4J). After METTL3 overexpression, precursor CARD11 expression levels were not affected, while mature CARD11 mRNA expression was increased (Fig. 4K‒L). The protein levels of CARD11 were also increased after METTL3 overexpression (Fig. 4M‒N). RIP assay confirmed the interaction between METTL3 and CARD11 (Fig. 4O). Additionally, METTL3 silencing decreased the m6A levels of CARD11, while METTL3 overexpression increased it (Fig. 4P). Through the SRAMP database, the authors obtained the two m6A modification sites (site 1#, 690; site 2#, 1769) with the highest degree of modification on the RNA sequence of CRAD11 (Fig. 4Q). Then, the luciferase activity of CRAD11 was decreased after METTL3 silencing, while site 1# mutation reversed it. At site 2#, the luciferase activity of CRAD11 showed no difference at the site 2# mutation type of wild type (Fig. 4R). Finally, the mRNA stability of CARD11 was prominently decreased after METTL3 silencing (Fig. 4S).

### CARD11 overexpression neutralized the effects of si-METTL3 on the osteogenic differentiation of hPDLSCs

**Fig. 5:**
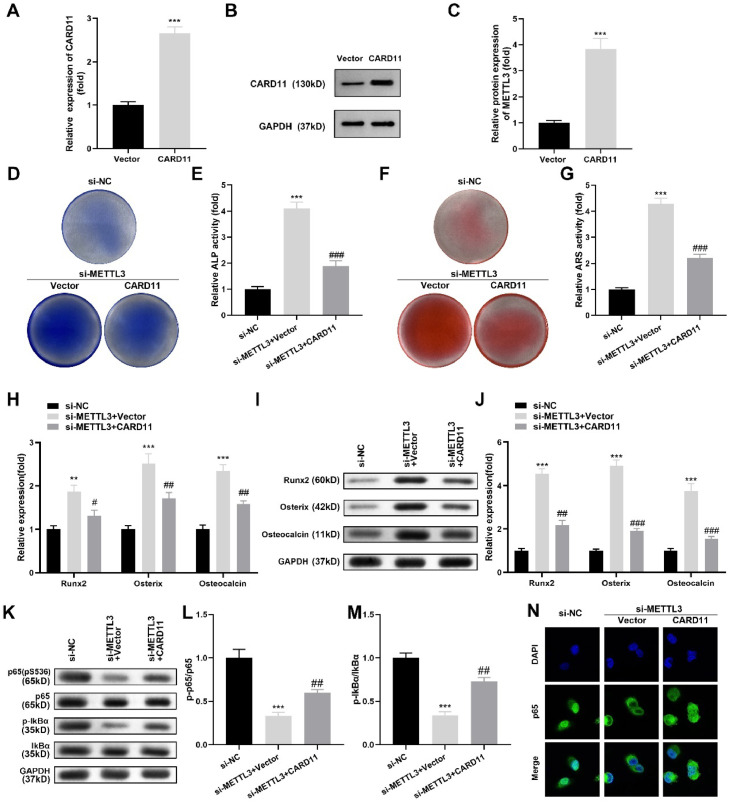
CARD11 overexpression neutralized the effects of si-METTL3 on the osteogenic differentiation of hPDLSCs. (A) RT-qPCR and western blotting (B‒C) were performed to validate the transfection efficiency. (D‒E) ALP activity of hPDLSCs. (F‒G) ARS staining of hPDLSCs. The levels of Runx2, Osterix and Osteocalcin were measured by qRT-PCR (H) and western blots (I‒J). (K‒M) The protein levels of p-p65 and p-IκBα were detected by western blotting. (N) The immunofluorescence assay was conducted to analyze the nuclear entry of P65 (n = 3). ** p < 0.01, *** p < 0.001, vs. the si-NC group. # p < 0.05, ## p < 0.01, vs. the si-METTL3+vector group.

Finally, the authors confirmed that the CARD11 overexpression vector prominently increased the mRNA (Fig. 5A) and protein (Fig. 5B‒C) levels of CARD11 in hPDLSCs. After CARD11 overexpression vector transfection, the authors found that ALP activity (Fig. 5D‒E) and red calcium nodules (Fig. 5F‒G) were prominently decreased in the si-METTL3-treated hPDLSCs. Additionally, Runx2, Osterix, and Osteocalcin were prominently downregulated at the mRNA (Fig. 5H) and protein (Fig. 5I‒J) levels in the si-METTL3-treated hPDLSCs after CARD11 overexpression vector transfection. Moreover, the authors found that METTL3 silencing prominently downregulated p-p65 and p-IƙBα protein expression in hPDLSCs, while the CARD11 overexpression vector neutralized the si-METTL3 effects (Fig. 5K‒M). Finally, the immunofluorescence assay showed that METTL3 silencing inhibited the nuclear entry of P65, while CARD11 overexpression promoted it (Fig. 5N).

### METTL3 knockdown relieved the periodontitis progression in vivo

**Fig. 6:**
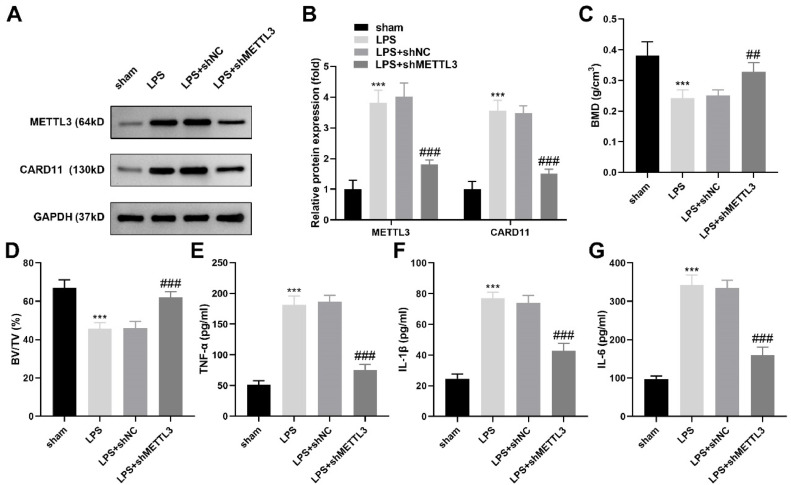
METTL3 knockdown relieved the periodontitis progression in vivo. (A‒B) The protein levels of CARD11 and METTL3 in the periodontal tissues of periodontitis rats were detected by western blot. The BMD (C) and BV/TV (D) levels were obtained by micro-CT analysis. The TNF-α (E), IL-1β (F) and IL-6 (G) levels in periodontal tissues of periodontitis rats were detected by ELISA kits (n = 3). *** p < 0.001, vs. the sham group. ## p < 0.01, ### p < 0.001 vs. the model + shNC group.

Finally, in periodontitis rats, the protein levels of CARD11 and METTL3 in periodontal tissues were significantly increased, while METTL3 knockdown significantly decreased them (Fig. 6A‒B). Additionally, METTL3 knockdown increased the BMD (Fig. 6C) and BV/TV (Fig. 6D) levels, and decreased the TNF-α (Fig. 6E), IL-1β (Fig. 6F), and IL-6 (Fig. 6G) in periodontal tissues of periodontitis rats.

## Discussion

Here, the authors demonstrated that METTL3 and CARD11 were overexpressed in periodontitis patients and that METTL3 was positively related to CARD11. Knockdown of MRTTL3 promoted the osteogenic differentiation of hPDLSCs by regulating CARD11 expression through the NF-κB signaling pathway.

Periodontitis is a chronic inflammatory disease characterized by gingival inflammation and alveolar bone resorption, which can cause progressive destruction of periodontal support tissue and irreversible resorption of alveolar bone, ultimately leading to tooth loss.^19^^,^^20^ Periodontal tissue includes alveolar bone, yayoumin, periodontal ligament, and cementum. There are a large number of PDLSCs in the periodontal ligament, which play an important role in the osteogenic balance of periodontal tissue.^21^ Since the microenvironment affects the behavior of stem cells at all times, scholars believe that alveolar bone resorption caused by periodontitis is closely related to changes in the osteogenic differentiation ability of PDLSCs.^22^, ^23^, ^24^ In this study, the authors first isolated and identified hPDLSCs. Through the detection of the positive markers CD90 (96.8 %), CD105 (98.2 %), and CD146 (97.4 %), as well as the negative markers CD11b (4.8 %) and CD45 (1.1 %), the authors confirmed that the cultured cells were stem cells.

Osteogenic differentiation is the key step of periodontal tissue repair and reconstruction. The expression of osteogenic markers by mineralization-induced hPDLSCs is also important evidence of their osteogenic properties.^25^ Recently, accumulating evidence has demonstrated that METTL3 plays an important role in osteogenic differentiation. For example, Yu et al.^26^ found that knockdown of METTL3 dramatically increased the calcium deposition and ALP activity of mesenchymal stem cells, suggesting an inhibitory role of METTL3 in osteogenic differentiation. However, currently, there is disagreement about the role of MELLT3 in osteogenic differentiation. Tian et al.^27^ demonstrated that MELLT3 was overexpressed during osteogenic induction of bone mesenchymal stem cells. Furthermore, after MELLT3 knockdown, bone formation-related genes, such as Runx2 and Osterix, were downregulated. The contradictory findings regarding the role of METTL3 in osteogenic differentiation may stem from differences in cell types, experimental conditions, and the complexity of METTL3′s molecular mechanisms. Similarly, the present study on hPDLSCs revealed that silencing METTL3 upregulated Runx2, Osterix, and Osteocalcin expression while increasing calcium deposition and ALP activity, supporting a pro-osteogenic function in this specific cell type. These discrepancies likely arise from variations in the biological properties of different stem cells (e.g., mesenchymal stem cells vs. bone mesenchymal stem cells vs. hPDLSCs) and potential differences in experimental setups or evaluation methods. Additionally, the multifaceted role of METTL3 as an m6A RNA methyltransferase may lead to context-dependent effects, highlighting the need for further investigation into its precise mechanisms in specific cellular contexts.

Subsequently, through bioinformatic analysis, the authors found that CARD11 was overexpressed in periodontitis patients. Knockdown of METTL3 decreased the m6A and mRNA levels of CARD11, which also indicated that CARD11 was positively regulated by MRTTL3. KEGG pathway analysis showed that CARD11 was enriched in the Nuclear Factor Kappa-B (NF-κB) signaling pathway. It was reported that TNF-α and lipopolysaccharide can activate the NF-κB signaling pathway by activating the IκK complex in dental pulp stem cells, which may be an important reason for interfering with the normal differentiation and tissue regeneration of inflammatory dental pulp stem cells.^28^ Many studies have confirmed that CARD11 can activate the NF-κB signaling pathway, which affects the progression of various diseases, such as primary CNS lymphoma,^29^ diffuse large B-cell lymphoma,^30^ and immunodeficiency disorder.^31^ Additionally, the NF-κB signaling pathway was reported to be involved in osteogenic differentiation. Ren et al.^32^ reported that inactivating the NF-κB pathway induced osteogenic differentiation. Chen et al.^33^ also demonstrated that progranulin accelerated the osteogenic differentiation of hPDLSCs by inhibiting NF-κB signaling. Similarly, this study found that overexpression of CARD11 reversed the effects of si-METTL3 on the osteogenic differentiation of hPDLSCs. Additionally, an increase in p-p65 and p-IκB was observed after CARD11 overexpression, which indicated that the NF-κB pathway was activated after CARD11 transfection.

However, there was still a limitation in this study. The GSE23586 dataset is outdated (2010). In the future, the authors will conduct more up-to-date bioinformatics analysis for continuity research.

In summary, this research demonstrated that METTL3 silencing decreased the m6A and mRNA expression of CARD11, thereby promoting the osteogenic differentiation of hPDLSCs by inactivating the NF-κB signaling pathway. These findings indicated that future clinical treatment of periodontitis can focus on the development of drugs targeting the METTL3 and CARD11 genes.

## Ethics approval and consent to participate

The study was approved by the Ethics Committee of the 900th Hospital of Joint Logistic Support Force ([2021]02–283–01). Written informed consent was obtained from all patients. All experiments were performed in accordance with relevant guidelines and regulations.

## Funding

This research did not receive any specific grant from funding agencies in the public, commercial, or not-for-profit sectors.

## Availability of data and materials

The datasets used and/or analyzed during the current study are available from the corresponding author on reasonable request.

## CRediT authorship contribution statement

**Bing Zhou:** Data curation, Formal analysis, Investigation, Resources, Software, Writing – original draft, Writing – review & editing. **Cheng Wang:** Conceptualization, Data curation, Methodology, Project administration, Writing – review & editing.

## Declaration of competing interest

The authors declare no conflicts of interest.

## Appendix Supplementary materials

Image, application 1

## References

[1] Slots J. (2017). Periodontitis: facts, fallacies and the future. Periodontol 2000.

[2] Bartold P.M. (2018). Lifestyle and periodontitis: the emergence of personalized periodontics. Periodontol 2000.

[3] Ribeiro A.B., Brognara F., Da S.J., Castania J.A., Fernandes P.G., Tostes R.C. (2020). Carotid sinus nerve stimulation attenuates alveolar bone loss and inflammation in experimental periodontitis. Sci Rep.

[4] Zhang X., Zhang S., Yan X., Shan Y., Liu L., Zhou J. (2021). M6a regulator-mediated rna methylation modification patterns are involved in immune microenvironment regulation of periodontitis. J Cell Mol Med.

[5] Larsson L., Thorbert-Mros S., Lopez-Lago A., Kalm J., Shikhan A., Berglundh T. (2016). Expression of tet2 enzyme indicates enhanced epigenetic modification of cells in periodontitis. Eur J Oral Sci.

[6] Lei F., Li M., Lin T., Zhou H., Wang F., Su X. (2022). Treatment of inflammatory bone loss in periodontitis by stem cell-derived exosomes. Acta Biomater.

[7] Lv P., Gao P., Tian G., Yang Y., Mo F., Wang Z. (2020). Osteocyte-derived exosomes induced by mechanical strain promote human periodontal ligament stem cell proliferation and osteogenic differentiation via the mir-181b-5p/pten/akt signaling pathway. Stem Cell Res Ther.

[8] Jia L., Zhang Y., Ji Y., Xiong Y., Zhang W., Wen Y. (2019). Yap balances the osteogenic and adipogenic differentiation of hpdlscs in vitro partly through the wnt/beta-catenin signaling pathway. Biochem Biophys Res Commun.

[9] He L., Li H., Wu A., Peng Y., Shu G., Yin G. (2019). Functions of n6-methyladenosine and its role in cancer. Mol Cancer.

[10] Chen Y.G., Chen R., Ahmad S., Verma R., Kasturi S.P., Amaya L. (2019). N6-methyladenosine modification controls circular rna immunity. Mol Cell.

[11] Lin Z., Hsu P.J., Xing X., Fang J., Lu Z., Zou Q. (2017). Mettl3-/mettl14-mediated mrna n(6)-methyladenosine modulates murine spermatogenesis. Cell Res.

[12] Liu P., Li F., Lin J., Fukumoto T., Nacarelli T., Hao X. (2021). M(6)a-independent genome-wide mettl3 and mettl14 redistribution drives the senescence-associated secretory phenotype. Nat Cell Biol.

[13] Tian C., Huang Y., Li Q., Feng Z., Xu Q. (2019). Mettl3 regulates osteogenic differentiation and alternative splicing of vegfa in bone marrow mesenchymal stem cells. Int J Mol Sci.

[14] Zhang Y., Gu X., Li D., Cai L., Xu Q. (2019). Mettl3 regulates osteoblast differentiation and inflammatory response via smad signaling and mapk signaling. Int J Mol Sci.

[15] Yamamoto-Furusho J.K., Fonseca-Camarillo G., Furuzawa-Carballeda J., Sarmiento-Aguilar A., Barreto-Zuniga R., Martinez-Benitez B. (2018). Caspase recruitment domain (card) family (card9, card10, card11, card14 and card15) are increased during active inflammation in patients with inflammatory bowel disease. J Inflamm (L).

[16] Meininger I., Krappmann D. (2016). Lymphocyte signaling and activation by the carma1-bcl10-malt1 signalosome. Biol Chem.

[17] Watt S.A., Purdie K.J., den Breems N.Y., Dimon M., Arron S.T., Mchugh A.T. (2015). Novel card11 mutations in human cutaneous squamous cell carcinoma lead to aberrant nf-kappab regulation. Am J Pathol.

[18] Li N., Li Z., Wang Y., Chen Y., Ge X., Lu J. (2021). Ctp-cm enhances osteogenic differentiation of hpdlscs via nf-κb pathway. Oral Dis.

[19] Cardoso E.M., Reis C., MC Manzanares-Cespedes (2018). Chronic periodontitis, inflammatory cytokines, and interrelationship with other chronic diseases. Postgr Med.

[20] Hoare A., Soto C., Rojas-Celis V., Bravo D. (2019). Chronic inflammation as a link between periodontitis and carcinogenesis. Mediat Inflamm.

[21] Tomokiyo A., Wada N., Maeda H. (2019). Periodontal ligament stem cells: regenerative potency in periodontium. Stem Cells Dev.

[22] Liu Y., Liu C., Zhang A., Yin S., Wang T., Wang Y. (2019). Down-regulation of long non-coding rna meg3 suppresses osteogenic differentiation of periodontal ligament stem cells (pdlscs) through mir-27a-3p/igf1 axis in periodontitis. Aging (Albany Ny).

[23] Chow A.T., Quah S.Y., Bergenholtz G., Lim K.C., Yu V., Tan K.S (2019). Bacterial species associated with persistent apical periodontitis exert differential effects on osteogenic differentiation. Int Endod J.

[24] Gan Z., Guo Y., Zhao M., Ye Y., Liao Y., Liu B. (2024). Excitatory amino acid transporter supports inflammatory macrophage responses. Sci Bull (Beijing).

[25] Zheng J., Zhu X., He Y., Hou S., Liu T., Zhi K. (2021). Circcdk8 regulates osteogenic differentiation and apoptosis of pdlscs by inducing er stress/autophagy during hypoxia. Ann N Acad Sci.

[26] Yu J., Shen L., Liu Y., Ming H., Zhu X., Chu M. (2020). The m6a methyltransferase mettl3 cooperates with demethylase alkbh5 to regulate osteogenic differentiation through nf-kappab signaling. Mol Cell Biochem.

[27] Tian C., Huang Y., Li Q., Feng Z., Xu Q. (2019). Mettl3 regulates osteogenic differentiation and alternative splicing of vegfa in bone marrow mesenchymal stem cells. Int J Mol Sci.

[28] Zaw S., Kaneko T., Zaw Z., Sone P.P., Murano H., Gu B. (2020). Crosstalk between dental pulp stem cells and endothelial cells augments angiogenic factor expression. Oral Dis.

[29] Montesinos-Rongen M., Schmitz R., Brunn A., Gesk S., Richter J., Hong K. (2010). Mutations of card11 but not tnfaip3 may activate the nf-kappab pathway in primary cns lymphoma. Acta Neuropathol.

[30] Knies N., Alankus B., Weilemann A., Tzankov A., Brunner K., Ruff T. (2015). Lymphomagenic card11/bcl10/malt1 signaling drives malignant b-cell proliferation via cooperative nf-kappab and jnk activation. Proc Natl Acad Sci U S A.

[31] Zhao P., Meng Q., Huang Y., Zhang L., Luo S., Zhang X. (2021). Identification and characterization of a germline mutation in card11 from a chinese case of b cell expansion with nf-kappab and t cell anergy. Front Immunol.

[32] Ren Y., Zhang K., Wang J., Meng X., Du X., Shi Z. (2021). Hotairm1 promotes osteogenic differentiation and alleviates osteoclast differentiation by inactivating the nf-kappab pathway. Acta Biochim Biophys Sin (Shanghai).

[33] Chen J., Yu M., Li X., Sun Q.F., Yang C.Z., Yang P.S. (2020). Progranulin promotes osteogenic differentiation of human periodontal ligament stem cells via tumor necrosis factor receptors to inhibit tnf-alpha sensitized nf-kb and activate erk/jnk signaling. J Periodontal Res.

