# Supplementary material for: METTL3 knockdown promotes the osteogenic differentiation of hPDLSCs by regulating CARD11 levels
Source: Clinics (Sao Paulo). 2025 Oct 14;80:100787. doi: 10.1016/j.clinsp.2025.100787 (PMC12550324; doi:10.1016/j.clinsp.2025.100787)

CLINICS-D-25-00275_Supplementary Material

**Supplementary Figure 1 si-METTL3 #1 transfection promoted the osteogenic differentiation of hPDLSCs.** (A‒B) ALP activity of hPDLSCs. (C‒D) ARS staining of hPDLSCs. (E) The mRNA levels of Runx2, Osterix and Osteocalcin were measured by Qrt-PCR. (F‒G) The protein levels of Runx2, Osterix and Osteocalcin were detected by western blotting (n = 3). ** p < 0.01, *** p < 0.001.


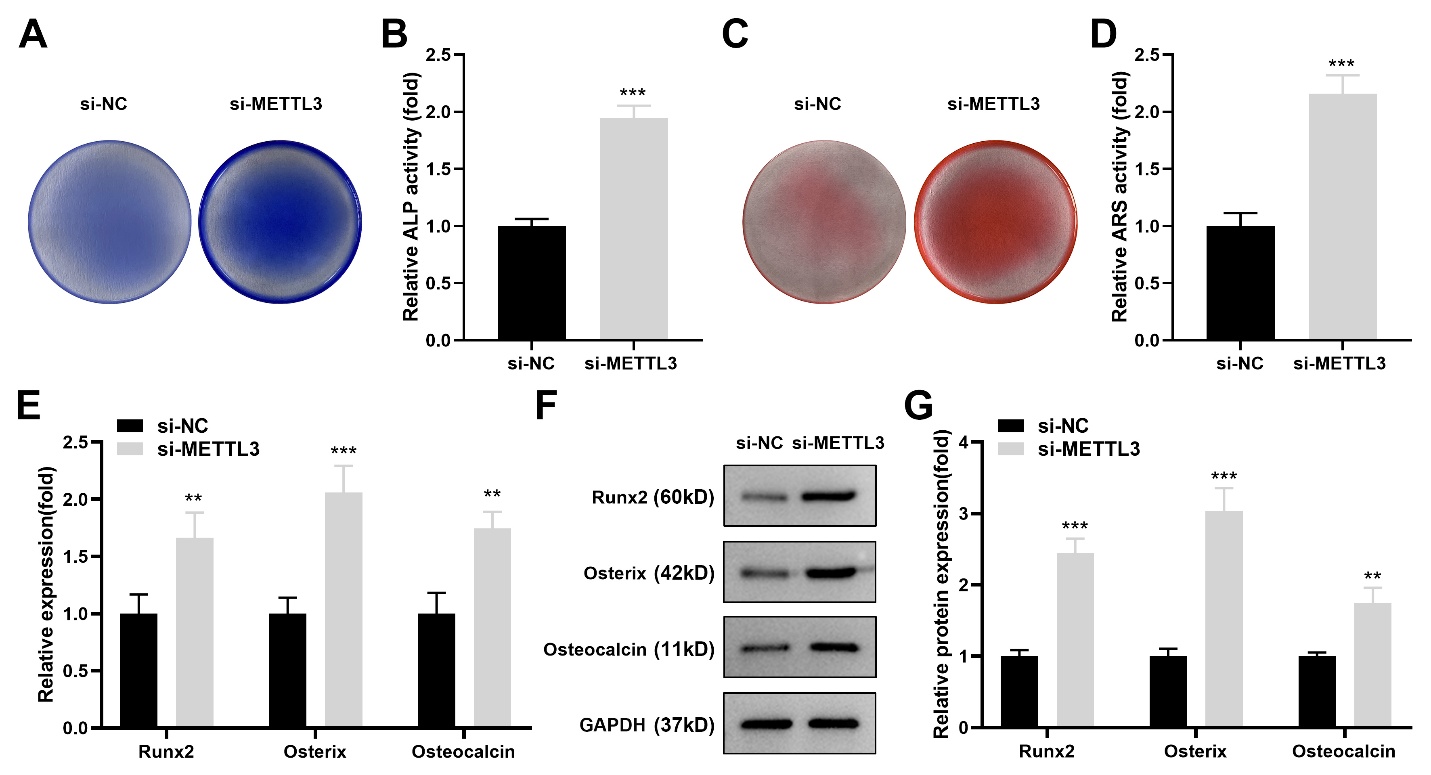

Supplement: Supplementary file 1 [file mmc1.docx]
